# Evaluation of the pediatric life support instructors courses

**DOI:** 10.1186/s12909-021-02504-2

**Published:** 2021-01-23

**Authors:** Jesús López-Herce, Angel Carrillo, Javier Urbano, Gema Manrique, y Santiago Mencía, y Grupo Madrileño de Cuidados Intensivos Pediátricos, Maria José Santiago, Maria José Solana, Jimena del Castillo, Rafael González, Jorge López, César Sánchez, Andrés Alcaraz, Amelia Sánchez, Sarah Fernández

**Affiliations:** 1 Servicio de Cuidados Intensivos Pediátricos, Hospital General Universitario Gregorio Marañón de Madrid, Dr Castelo 47, 28009 Madrid, Spain; 2 Departamento de Salud Pública y Maternoinfantil, Facultad de Medicina, Universidad Complutense de Madrid, Madrid, Spain; 3 Instituto de Investigación Sanitaria del Hospital Gregorio Marañón, Madrid, Spain; 4 Red de Salud Maternoinfantil y del Desarrollo (RedSAMID). RETICS financiada por el PN I+D+I 2008-2011, ISCIII - Subdirección General de Evaluación y Fomento de la Investigación y el Fondo Europeo de Desarrollo Regional (FEDER), ref. RD12/0026, Madrid, Spain; 5 Grupo Español de Reanimación Cardiopulmonar Pediátrica y Neonatal, Madrid, Spain

**Keywords:** Cardiopulmonary resuscitation, Cardiopulmonary resuscitation instructors, Children, Pediatric cardiopulmonary resuscitation instructors

## Abstract

**Objective:**

To evaluate the results and quality of pediatric cardiopulmonary resuscitation (CPR) instructor training courses.

**Methods:**

A retrospective analysis was performed of the results of 24 pediatric CPR instructor courses held over 21 years (1999 to 2019). The results of participants’ evaluation of theory and practice sessions were analyzed. In addition, participants were asked to answer an anonymous survey to assess their opinion on the quality of theory and practice lessons, course organization and methodology, and instructor training. The results were compared by professional groups.

**Results:**

A total of 560 participants completed the instructor course. Of them, 554 passed theory and practice tests (98.9 %). The mean score obtained in theory tests was 9.2 (0.8) out of 10. The mean score obtained in all practice tests was > 3.5 out of 5. Participants evaluated all the aspects of the course (theory and practice content, organization, teaching methodology, and instructors) with mean scores over 8 out of 10.

**Conclusions:**

Specific pediatric and neonatal CPR instructor courses are a cornerstone in the process of CPR training and ensuring the homogeneity and quality of training. Most of the participants obtained the qualification of instructors and their evaluation of the course was very positive.

## Background

Training both, healthcare professionals and the general public in cardiopulmonary resuscitation (CPR) is essential to improve CPR outcomes and the prognosis of patients suffering a cardiac arrest [1–3].

CPR training involves different phases: a study of training needs, design of the training process, selection of teaching methods, programming of teaching activities, training of instructors, implementation of training activities and evaluation of learning outcomes (results of the participants and quality of training), and the impact of the training process on clinical practice [1–4].

In order to achieve a high-quality training process, the main scientific organizations involved in CPR have designed training courses for instructors. Some have opted for a generic course for adult or pediatric CPR instructors [1, 3, 5], while others have designed specific instructor courses for each type of CPR [2, 6, 7].

Accordingly, evaluating participants’ performance and the quality of training is essential to improve CPR outcomes.

Many studies have analyzed the learning outcomes of participants in adult and pediatric CPR courses. However, despite their importance, very few studies have analyzed outcomes of instructor training or evaluated the quality of training in instructor courses [6], although it has been observed that poor learning outcomes occur because instructors are not adequately trained [8–10].

Some groups recommend that adult CPR instructor courses are only offered to physicians, whereas pediatric CPR instructor courses should only be aimed at pediatricians. Nevertheless, the policy of the Spanish Pediatric and Neonatal Resuscitation Group (SPNRG) is to allow access to all health professionals who have passed the Pediatric Advanced Life Support (PALS) course and have experience in pediatric CPR.

The aim of our study was to analyze learning outcomes and the quality of pediatric and neonatal CPR instructor courses and compare the results obtained by professional groups.

## Methods

### Study design

A retrospective analysis of the results of pediatric and neonatal CPR instructor courses given between 1999 and 2019 and certified by the SPNRG was carried out.

### Participants

The minimum requirements for the course were to have passed a SPNRG-certified PALS course and having clinical experience in pediatric and neonatal CPR. Participants were selected by the director of the course according to their previous training and experience in CPR and teaching, as well as to the educational needs of their professional area.

The number of participants ranged from 20 to 36, depending on the classrooms and instructors available, with a maximum number of six participants per practice group.

All instructors were SPNRG-certified PALS instructors.

All participants gave consent for participation in the study and publication of results.

### Methodology

The methodology of the course is described elsewhere (6). The duration of the course was 26–28 h distributed over 3–4 days.

The course is divided into 2 phases: an initial preparation phase and a phase involving face-to-face sessions.

- In the initial phase, participants receive the Guide for Instructors and were given instructions to prepare for each practice session. In this phase, doubts are resolved by distance communication with the director of the course.
- The face-to-face phase consists of theory and practice sessions. Theory sessions (i.e. instructor course, organization of pediatric life support courses, teaching methods, preparation of clinical cases and practice sessions, and evaluation methodology) have a duration of 6 hours and are developed interactively, stimulating the participation of students. Practice sessions (i.e. public speaking, basic life support, airway ventilation, venous and intravenous access, arrhythmias, trauma and CPR, neonatal resuscitation, and integrated advanced resuscitation) have a duration of 20 hours. In practice sessions, the instructor first explains the material and scenario and the guide-model of practice and then each participant plays the role of instructor, while the other participants act as if they were attending a PALS course. After each performance, the participant performs self-assessment and, afterwards, the whole group analyzes how the student managed the session (i.e. presentation of the clinical case, development of the practice class, and student evaluation). Finally, the instructor makes a summary. Positive feedback is used to build up a climate of friendship and confidence within the group.

In the public speaking practice, each student gives an oral presentation using four or five slides from PALS courses which they had received in advance. After each presentation, the participant, their colleagues, and the instructor analyze the quality of the presentation, the capacity to catch and maintain attention, clarity, oral expression, and body language, among other elements.

Over time, some modifications have been made to the program, according to the evaluations made by the participants and instructors of the course, progressively increasing the workload of participants prior to the course.

### Evaluation

- Theoretical evaluation:

The final theory test consisted of 20 items, including multiple-choice questions with 5 options and sequential-order tasks. Theory tests were scored over a 10-point scale.

b) Practical evaluation:

The performance of each participant as an instructor at each practice session was evaluated, and an overall score ranging from 1 to 5 was obtained according to the criteria shown in Table 1.

**Table 1:** Evaluation criteria for the practice sessions

Practice of evaluation sheet of the instructor course		
Practice	Instructor	Date
- Presentation and objectives		
- Guide model		
- Planning and monitoring of the simulation		
- Evaluation		
- Overall impression		
**Practice of oral expression techniques**		
Instructor:	Date	
- Presentation and objectives		
- Content		
- Verbal expression		
- Gestural expression		
- Overall impression		
**Scoring**		
1: The student does not do it		
2: The student does it wrong		
3: The student does it with some defects, but ends up doing it properly		
4: The student does it well, with some minor flaws		
5: The student does it very well		
The overall impression should reflect the ability of the student to lead practice as an instructor		

Theory and practice evaluations and the evaluation criteria were previously validated by the Scientific Committee of the SPNRG.

iii) Final evaluation of the students.

At the end of the course, the instructors evaluated theory and practice test results and judged whether the participants had reached sufficient level to merit the instructor diploma. To pass the course, participants were required to have a minimum score of 6.5 in theory tests, and a mean score higher than 3.5 in practice tests. A score < 3 was not allowed in more than two practice evaluations.

iv) Evaluation of the course by participants.

At the end of the course, participants filled in an anonymous questionnaire aimed at assessing the quality of the course, including individual evaluations of each theory and practice session, different aspects of organization, methodology and evaluation of instructors, with a score from 0 to 10.

### Statistical methods

Statistical analysis of the results was performed with SPSS version 20 software (SPSS Inc, Chicago, USA). Continuous variables are expressed as mean values and standard deviations, whereas categorical variables are expressed as frequencies and percentages. The chi-square test was used to compare categorical variables. For the comparison of the scores in the different professional categories (we compared all together and one per one with the rest of categories) Student’s *t-*tests and ANOVA tests were applied, with Bonferroni correction or Games-Howell tests based on the homogeneity of variances. For comparison of theory and practice scores, the ANOVA test of repeated measures with Bonferroni correction was used. A *p* value lower than 0.05 was considered statistically significant.

## Results

Twenty-four instructor courses involving 560 participants were analyzed. Participant distribution by professional group is shown in Table 2. Most of the participants were hospital pediatricians and pediatric residents.

**Table 2:** Student distribution by professional groups

	Number	Percentage
Hospital pediatrician	248	44.3
PICU pediatrician	62	11.1
Primary care pediatrician	16	2.9
Pediatric resident	121	21.6
Nurse	54	9.6
Emergency physician	27	4.8
Others	2	0.4
Unknown	30	5.4
Total	560	100

*PICU* pediatric intensive care unit

### Results of the participants

Table 3 summarizes the global results obtained in theory and practice tests, with comparisons by professional group and workplace.

**Table 3:** Scoring of theory and practice sessions (mean and standard deviation)

	Global	Hospital pediatrician	PICU pediatrician	PC pediatrician	Pediatric resident	Nurse	Emergency physician	P
Theory evaluation	9.2	9.3	9.4	9.5	9.4	9.2	9.1	.252
	0.8	0.8	0.7	0.4	0.9	0.9	0.8	
Expression techniques	3.8	3.8	3.8	3.7	3.8	3.6	3.8	.287
	0.6	0.6	0.6	0.7	0.7	0.6	0.5	
Basic CPR	3.8	3.7	3.9	3.6	3.8	3.6	3.7	.013
	0.6	0.6	0.6	0.6	0.5	0.6	0.5	
Airway	3.8	3.7	4.0	3.5	3.9	3.7	3.6	.009
	0.6	0.7	0.7	0.7	0.6	0.6	0.6	
Vascular accesses	3.9	3.8	4.1	3.8	3.9	4.1	3.9	.023
	0.6	0.6	0.6	0.7	0.7	0.6	0.6	
Arrythmias	3.6	3.5	3.8	3.2	3.7	3.5	3.7	.036
	0.7	0.7	0.7	0.8	0.7	0.8	0.7	
Trauma and CPR	3.6	3.6	3.8	3.2	3.6	3.5	4.1	.012
	0.7	0.7	0.7	0.5	0.7	0.8	0.5	
Neonatal CPR	3.9	3.9	3.9	3.8	4.0	3.5	3.7	.000
	0.6	0.6	0.7	0.7	0.6	0.8	0.7	
Integrated CPR	3.8	3.7	4.0	3.4	3.8	3.6	3.7	.000
	0.6	0.7	0.6	0.7	0.6	0.6	0.7	

*CPR* cardiopulmonary resuscitation, *PC* primary care, *PICU* pediatric intensive care unit

Overall, 98.9 % of all participants achieved a positive evaluation and obtained the instructor diploma. The 6 participants who did not obtain the diploma were offered to repeat the instructor course, and one did so.

Regarding theory tests, no significant differences were observed in student scores based on their professional group.

There were no significant differences in the scores obtained in the different practice tests in the global evaluation where all participants were included. Participants of all occupations obtained scores ≥ 3.2 in each practice. Individual evaluation of each practice session showed non-relevant but statistically significant differences.

Pediatric intensive care unit (PICU) pediatricians obtained slightly higher scores than the rest of professionals in most practices, although differences were not statistically significant.

When each professional professional group was compared with the rest, emergency physicians obtained a higher mean score in trauma practice, but a significant difference was only observed in comparison with primary care pediatricians (*p* = 0.034).

In neonatal practice, PICU pediatricians, hospital pediatricians and pediatric residents scored higher than nurses, but differences were only significant in the case of hospital pediatricians (*p* = 0.002) and pediatric residents (*p* = 0.001).

In the practice of integrated advanced CPR, PICU pediatricians scored significantly higher than hospital pediatricians (*p* = 0.018), nurses (*p* = 0.005) and primary care pediatricians (*p* = 0.006).

### Course evaluation survey by the participants

Table 4 shows the overall evaluation and comparison by professional group of organization, teaching methodology, instructor skills, and theory and practice sessions of the 540 survey of the students.

**Table 4:** Evaluation of teaching methodology, teaching staff and organization

	Mean	SD
**Organization**		
Previous information	8.46	0.09
Documentation	8.62	0.07
Organization	9.12	0.05
Time schedule	8.18	0.08
Place	8.12	0.11
Material	8.66	0.05
Time	8.46	0.06
**Methodology and instructors**		
Meeting objectives	9.18	0.04
Methodology	8.89	0.05
Structuring of contents	8.82	0.07
Knowledge of the subject	9.42	0.04
Clarity of exposition	9.15	0.05
Arise interest	9.13	0.05
Stimulate participation	9.23	0.05
Create a climate of trust	9.08	0.09
**Theory classes**		
Instructor course	8.37	0.18
Organization	8.66	0.36
Updates in CPR	8.89	0.76
Teaching techniques	8.97	0.08
Preparation of classes	8.56	0.97
Evaluation	8.50	0.09
**Practices**		
Basic CPR	8.48	0.18
Airway	8.59	0.36
Vascular accesses	8.70	0.76
Arrhythmias	8.58	0.08
Trauma	8.46	0.97
Neonatal	8.91	0.09
Advanced CPR	9.03	0.18
Expression techniques	9.12	0.36

*SD* standard deviation, *CPR* cardiopulmonary resuscitation

- Organization, teaching methodology and teaching staff: All aspects of organization and teaching methodology were scored above 8. The least valued parameters were the venue, time schedules and time given to develop the contents of the course. All items regarding the teaching staff received a mean score above 8.8. Most participants feel that, although the course is long, the duration of practice sessions should be prolonged to be able to practice as instructors in all modalities.
- Theory and practice sessions: All theory and practice sessions were evaluated with a mean score above 8.3, although there were significant differences in the evaluation of the different theory classes (*p* < 0.001) and practice sessions (*p* < 0.001). The theory sessions about updates in CPR and teaching techniques and the practice sessions on integrated advanced CPR and expression techniques were the most valued.

## Discussion

This is the first study to extensively analyze the results of pediatric CPR instructor courses and assess participants’ opinion about the course received, over a long period of time with a significant number of participants.

### Course methodology

The practice methodology of the instructor course is a crucial aspect and is primarily based on simulation [11]. The participants are the protagonists since they are the ones who develop the practices, evaluate themselves and participate in peer-assessment. The instructors act as facilitators and advisors, and direct and organize the practice session so that it reproduces a practice from a PALS course. Instructors also facilitate that conclusions are draw by all participants. Public speaking practices help participants improve their communication skills and solve problems related to communication and group management.

Some institutions, such as the European Resuscitation Council (ERC), consider that a general instructor course is sufficient to train professionals in teaching any type of CPR course [1, 3, 5]. However, the SPNRG considers that instructors should be trained in each practice session of the pediatric CPR course [6], and the results of our study and the participants’ opinion support this view.

Practice sessions related to neonatal and integrated resuscitation were the most highly rated by participants, perhaps because in these practices they can best perform all instructor functions and manage a group. Although these practices are the most complex, they allow a better assessment of the ability of the instructor to teach CPR in an integrated manner.

An essential aspect of the methodology is the use of structured and supported debriefing [12]. In debriefing, correction and positive feedback are very important. Analyzing mistakes is the best way to improve our competence, correcting performance without making the student feel personalized. Self-assessment and peer-assessment help create a climate of trust that favors the acceptance of feedback and helps participants learn this methodology.

### Results of the course

Our results show that a specific pediatric CPR instructor course focused mainly on practice simulation methods is adequate for instructor training, since 98.9 % of participants achieved the learning objectives.

Most participants passed the course, which confirms its usefulness and the fact that a good selection of candidates had been previously made. A large proportion of participants were hospital pediatricians, including PICU and senior pediatric residents. Most nurses worked in PICUs and other special units and had extensive experience in pediatric CPR. Emergency care physicians were professionals who performed CPR in their regular practice.

Our study shows that students receive sufficient global training as pediatric CPR instructors. However, some of the professional groups them (i.e. primary care nurses and pediatricians) have more difficulties in certain practices, such as in the management of arrhythmias, CPR, and trauma, and neonatal CPR in the case of nurses, or advanced CPR in the case of primary care pediatricians. This is likely because they have less clinical experience in such tasks, which reinforces the relevance that instructors are experts in the subject they teach and the fact that not all instructors can teach all practices.

The training provided in instructor courses is necessary, but not sufficient. That is why the SPNRG requires that instructors who pass the course be supervised by expert instructors for at least two PALS courses over the next two years to reinforce training. Participants’ feedback confirms this view. On the other hand, in the long term, periodic re-training and updating of instructors is necessary to maintain their competence.

### Evaluation of the course by participants

The evaluation of the course by participants is a cornerstone of course quality control. This process serves to detect weaknesses and propose modifications in the organization, methodology and teaching staff for successive editions, in addition to integrating the participants in the training process [13].

The participants rated very positively organizational and methodological aspects, theory and practice sessions, and the teaching skills and attitude of instructors. Some aspects of the organization along with time schedule were among the worst rated aspects of the course. Due to the long duration of the course, the face-to-face phase has a duration of 26 to 28 h, sessions are very long, which increases participants’ fatigue and reduces their learning capacity. Thus, it is not surprising that this aspect was the worst rated. On the other hand, participants consider very important not to reduce the number of practice hours of the course and that all participants play the role of instructor in all practice sessions. Therefore, the practice phase cannot be replaced with distance training.

Taking into account that it is increasingly easier to offer distance learning through digital platforms, it is necessary to consider reducing the duration of the theory part of the course in favor of practice sessions. However, we consider that theory should not be completely eliminated because interaction between the instructor and the student in theory sessions is also essential.

It is worth noting the excellent evaluation of instructor teaching skills, coordination and attitude, which supports the usefulness of the training system for pediatric CPR instructors developed by the SPNRG.

### Limitations

Our study has some limitations. In the first place, it is a single-center study, which favors the homogeneity of the results; therefore, other training groups should confirm our results. However, the number of courses analyzed, the variety of professions of participants, and the venues where the course took place allow us to assume that the sample is representative of all the courses given in our country.

On the other hand, our study only analyzes the opinions of participants. In order to have a more comprehensive evaluation, it would have been necessary to analyze the opinions of instructors as well.

Finally, the satisfaction survey of participants did not correlate with the results obtained in the course because the survey was anonymous. Nevertheless, since the vast majority of participants passed the course, it is likely that this fact is not a critical factor in the evaluation of the course. The performance of participants as simulated instructors was not analyzed.

## Conclusions

We conclude that a specific pediatric and neonatal CPR instructor course focused mainly on practice simulation methods is an adequate method for training health professionals to teach pediatric resuscitation and it is well valued by the students. Our experience and methodology can be useful for other teaching groups [14–16].

This course is adequate to different health professional groups but some of the them have more difficulties in certain practices.

The involvement of all participants in all practice sessions reinforces learning, since participants mainly learn by acting and correcting themselves.

## Data Availability

Anonymous data of the results of the evaluations could ask for the authors.

## Abbreviations

CPR: Cardiopulmonary resuscitation
ERC: European Resuscitation Council
PALS: Pediatric Advanced Life Support
PICU: Pediatric intensive care unit
SPNRG: Spanish Pediatric and Neonatal Resuscitation Group
